# Spatial and temporal resource partitioning in a mixed‐species colony of avian echolocators

**DOI:** 10.1002/ece3.9805

**Published:** 2023-02-16

**Authors:** Keren R. Sadanandan, Hui Zhen Tan, Hong Yao Lim, Yi Gain Tan, Grace Lee, Lena Chan, Yifan Pei, Frank E. Rheindt, Maude W. Baldwin

**Affiliations:** ^1^ Evolution of Sensory Systems Research Group Max Planck Institute for Biological Intelligence Seewiesen Germany; ^2^ Department of Biological Sciences National University of Singapore Singapore Singapore; ^3^ Sentosa Development Corporation Singapore Singapore; ^4^ International Biodiversity Conservation Division National Parks Board of Singapore Singapore Singapore; ^5^ Department of Behavioural Ecology and Evolutionary Genetics Max Planck Institute for Biological Intelligence Seewiesen Germany

**Keywords:** bioacoustics, breeding seasonality, cave‐nesting, colony nesting, phenology

## Abstract

Resource partitioning may facilitate the coexistence of sympatric species with similar ecological requirements. Here, we study a colony of unusual echolocating birds called swiftlets, which nest underground on an island off the coast of Singapore. The colony comprises two congeneric swiftlet species, black‐nest swiftlets (*Aerodramus maximus*) and edible‐nest swiftlets (*A. fuciphagus*), nesting at high densities and in close proximity. Bioacoustic recordings and monitoring of nesting biology at the site across multiple seasons revealed significant differences in echolocation calls as well as survival rates between the species, with the larger black‐nest swiftlet nesting at locations with the highest fledging rates. We also observe an additional off‐season breeding peak by the smaller species, the edible‐nest swiftlet. Unexpectedly, off‐season egg‐hatching rates were significantly higher compared with the rates during the shared breeding season (mean difference = 14%). Our study on the breeding biology of these echolocating cave‐dwelling birds provides an example of spatial and temporal strategies that animals employ to partition resources within a confined habitat.

## Graphical abstract

Swiftlets are unusual birds that nest in dark caves and can navigate using echolocation. We study an underground colony of 2 congeneric swiftlets and find differences in their life history strategies, echolocation calls, and offspring survival rates. We document interspecific spatial segregation of nests as well as staggered breeding seasonality between both species.

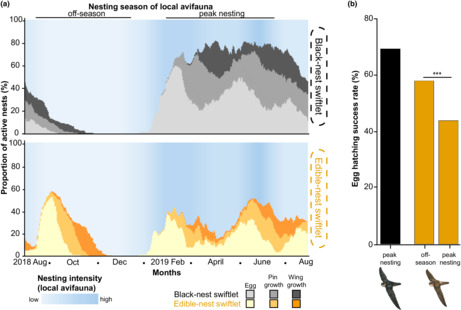

## 1 INTRODUCTION

Niche theory predicts that co‐occurring species in a community occupy different niches in order to minimize overlap in resource use (Hutchinson, 1991; MacArthur & Levins, 1967; Schoener, 1974). Behavioral and morphological diversity can evolve to facilitate resource partitioning, as seen in radiations of *Anolis* lizards and Darwin's finches (Grant & Grant, 1979; Pacala & Roughgarden, 1982). Classical examples of resource partitioning have been described in colony‐nesting species. Colonial nesting—the nesting of large numbers of individuals (and often of multiple species) in a single location—occurs frequently in some bird groups, such as seabirds (Brown & Brown, 2001; Navarro et al., 2013; Oro et al., 2009). When nest sites are limited, individuals have to nest in close proximity, and the costs of over‐crowding include increased ectoparasite and disease transmission (Brown & Brown, 2001). Birds are sometimes known to spatially segregate in these colonies, or to stagger their breeding seasons, in order to successfully share a relatively small breeding site (spatial and temporal partitioning respectively; Bretagnolle et al., 1990; Brown et al., 2015; Burger & Shisler, 1978; Hunter, 1983; Monteiro & Furness, 1998; Navarro et al., 2013; Oro et al., 2009).

Colonial nesting also occurs in caves, where space is limited—cave nesters include many species of bats and most members of a radiation of birds called swiftlets (genus *Aerodramus*). Swiftlets are diurnal aerial insectivores comprising over 20 species that occur on islands in the Indian Ocean to the South Pacific (Rheindt et al., 2014). They belong to a large avian assemblage called Strisores, which contains specialized and diverse taxa such as hummingbirds (Trochilidae) and nightjars (Caprimulgidae). Swiftlets are an enigmatic group of birds that possess two highly specialized physiological faculties—echolocation and the ability to produce a sticky salivary substance for nest building—that enable them to navigate in dark caves and build nests that can adhere to cave walls (Lim & Earl of Cranbrook, 2014). The ability to echolocate has evolved only a handful of times in the animal kingdom, mostly in mammals, including bats and dolphins (Leonard & Fenton, 1984; Norris et al., 1961). Avian echolocation, which is poorly studied compared with mammalian echolocation, is found only in *Aerodramus* swiftlets and allies as well as in one distantly related bird lineage, the oilbird (*Steatornis caripensis*), from South America (Brinkløv et al., 2013). Similarly, although saliva is incorporated into nests by other members of the swift family, its use as the primary nest building material is unique to *Aerodramus* swiftlets.

**FIGURE 1:**
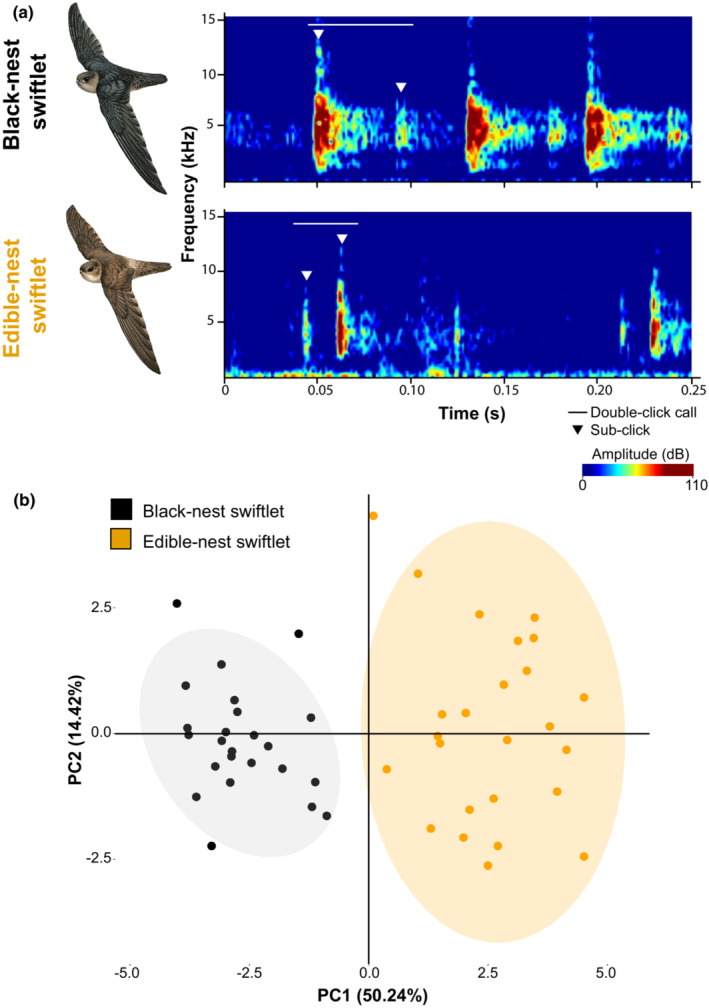
Echolocation calls differ between black‐nest (*Aerodramus maximus*) and edible‐nest swiftlets (*A. fuciphagus*). (a) Sound spectrograms of double‐click echolocation calls produced by black‐nest and edible‐nest swiftlets; white triangles indicate sub‐clicks and horizontal white lines indicate a double‐click call. See also Figure A1. Swiftlet illustrations are reproduced with permission of Lynx Edicions. (b) Principal component analysis of 16 vocal parameters (derived from 49 unique recordings) measured for both species, with ellipses representing 95% confidence intervals of the principal component (PC) scores for each species. PC1 accounted for 50.24% of total variance and was positively correlated with minimum frequency of motifs, duration of interval between sub‐clicks, and maximum and minimum frequencies within the recording. PC2, accounting for 14.42% of total variance, was positively correlated with peak and center frequency, difference in maximum frequency between the first and second sub‐clicks, and the duration of the first sub‐click.

Two widespread colonial members of *Aerodramus* are the black‐nest swiftlet (*Aerodramus maximus*) and the edible‐nest swiftlet (*A. fuciphagus*) (Figure 1a). Black‐nest swiftlets owe their name to the nests they construct, which are comprised of a mix of saliva and their own black body feathers, giving the nests a black or gray appearance. The nests of edible‐nest swiftlets are constructed entirely of saliva (therefore appearing white) and are regarded as a culinary delicacy in eastern Asia (Marcone, 2005). Both swiftlets have a largely overlapping range across Sundaland, and are often observed nesting together in caves (Eaton et al., 2021; Rheindt et al., 2014).

Although studies on resource partitioning in other colonial cave‐nesting species, such as bats, have primarily focused on differences related to foraging guilds and echolocation calls (Andreas et al., 2013; Heller & Helversen, 1989; Nicholls & Racey, 2006), few have documented differences in breeding strategies related to spatial and temporal partitioning. Here, we monitor a dense, mixed colony of black‐nest and edible‐nest swiftlets over the course of a year to understand their breeding biology. We quantify and diagnose differences in echolocation calls, analyze the timing of hatching and chick development to quantify their relative rates of reproductive success, and investigate whether any spatial segregation or temporal staggering of breeding seasonality occurs between these species. Our findings furnish unique insights into both behavioral differences and resource partitioning between two highly specialized and ecologically similar species.

## 2 MATERIALS AND METHODS

### 2.1 Study species and site

We studied a mixed colony of black‐nest and edible‐nest swiftlets on Sentosa, an island off the southern coast of Singapore. Located in an abandoned World War II underground bunker, the colony is the largest of its kind in Singapore. As the site is currently inaccessible to the public, it receives minimal disturbance. The first swiftlet nests in this bunker were recorded in 1987, when about 120–150 were reported (Lim, 2009); since then, the number of nests in the colony has doubled. Swiftlets are nonmigratory, and use this site throughout the year for nesting and roosting.

The bunker consists of a series of narrow corridors with two entrances, one at each end. We monitored light levels throughout the structure (details below), confirming that all corridors are in almost total darkness except for limited sections, such as hallways near the entrances, which receive some light. As the ceilings are consistently <3 m high—much lower than the natural caves favored by *Aerodramus*, which can extend to over 100 m in height (Lim & Earl of Cranbrook, 2014)—birds nest between 2 and 2.5 m above the floor, allowing for close observation. Despite the reduced elevation as compared to natural cave environments, the bunker appears to be a suitable breeding site, having been colonized spontaneously by swiftlets and actively used for more than 30 years.

### 2.2 Bioacoustics of echolocation calls

We recorded swiftlet echolocation calls (human‐audible clicks with a peak frequency range of 2–8 kHZ) during visits to the bunker between August 2018 and August 2019. During the nesting season, male and female swiftlets take turns incubating their eggs or young during the day (Lim & Earl of Cranbrook, 2014). We obtained sound recordings of individual birds of each species when they were flushed from incubating their egg(s) or young in a numbered nest, thus allowing us to assign species identity with certainty. The echolocating calls were recorded using a Sennheiser MKE 600 microphone and an Olympus LS‐12 Linear PCM recorder. Each recording was tagged with its nest number. Only recordings with unique nest numbers were used in subsequent analyses to ensure that each recording represents a different individual. Recordings of insufficient quality in which motifs of echolocation clicks were unclear or affected by background noise were discarded. We recorded double‐click calls from both black‐nest (*n* = 24) and edible‐nest swiftlets (*n* = 25), and single‐click calls only from black‐nest swiftlets (*n* = 25).

**FIGURE A1:**
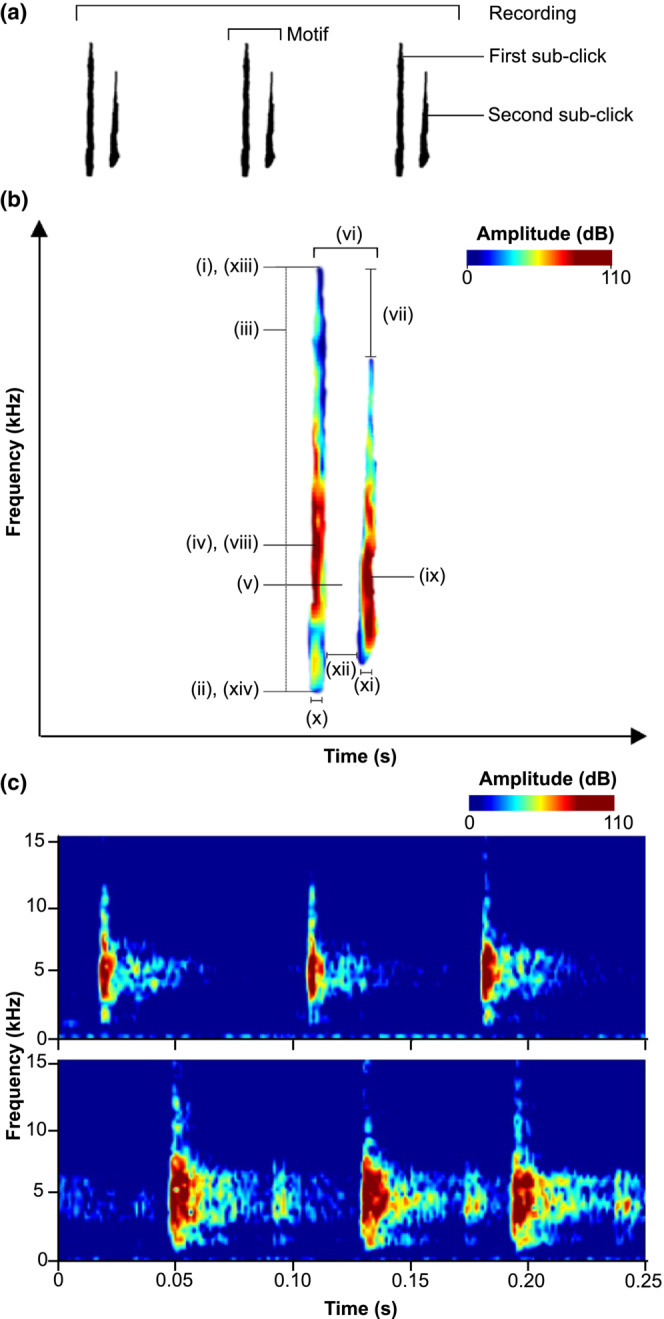
Echolocation call spectrograms. (a) Trace from a typical bioacoustic recording from a black‐nest swiftlet comprising 3 echolocation calls. (b) Echolocation call spectogram labelled with parameters measured for the bioacoustic analysis, including (i) maximum frequency of each motif, (ii) minimum frequency of each motif, (iii) frequency range of each motif, (iv) peak frequency of each motif, (v) center frequency of each motif, (vi) average number of sub‐clicks that each motif has within a recording, (vii) difference in maximum frequency between the first and second elements, (viii) peak frequency of the first element, (ix) peak frequency of the second element, (x) duration of the first element, (xi) duration of the second element, (xii) duration of the interval between the first and second element, (xiii) maximum frequency of each recording, and (xiv) minimum frequency of each recording. (c) Examples of the two call types emitted by black‐nest swiftlets, the single‐click call (upper) and the double‐click call (lower; same as in Figure 1).

The recordings were viewed and analyzed using Raven Pro Version 1.5 (Bioacoustics Research Program, Cornell Laboratory of Ornithology) (window size = 130; other settings at default values). Across the 49 double‐click recordings, a total of 317 motifs were analyzed using a standardized protocol (Rheindt et al., 2020; Sin et al., 2022). Sixteen parameters were measured for each motif (Figure A1b): (i) maximum frequency of each motif, (ii) minimum frequency of each motif, (iii) frequency range of each motif, (iv) peak frequency of each motif (the frequency when the motif is at its maximum power), (v) center frequency of each motif (median frequency), (vi) average number of sub‐clicks that each motif has within a recording, (vii) difference in maximum frequency between the first and second elements, (viii) peak frequency of the first element, (ix) peak frequency of the second element, (x) duration of the first element, (xi) duration of the second element, (xii) duration of the interval between the first and second element, (xiii) maximum frequency of each recording, (xiv) minimum frequency of each recording, (xv) ratio of maximum amplitude of the first element to the second, and (xvi) ratio of peak amplitude of the first element to the second. For the single‐click calls, we measured parameters (i)–(vi).

The 16 measured parameters of calls with two elements were analyzed using principal component analysis (PCA) conducted using R version 3.5.2 (R Core Team, 2021) to determine if the calls of the two swiftlet species are distinct from each other. The function “prcomp” was used to construct the PCA model, standardizing the input data to a zero mean and a variance of one. The parameters were also analyzed using a conservative vocal diagnosability criterion (the Isler criterion; Isler et al., 1998), which has been widely applied to distinguish between vocalizations of diverse pairs of closely related bird species (Cros & Rheindt, 2017; Gwee et al., 2019).

### 2.3 Nesting ecology

We visited the swiftlet colony in 2018 and mapped the interior. Each wall in the bunker was labeled and each nest on every wall was given a unique identifier. The location of each nest was recorded using Open Source QGIS (QGIS Development Team, 2022), and the distance of each nest from the nearest entrance was measured. Detailed observations on egg laying and chick development were collected from these nests across 71 visits over a period of 12 months in order to monitor nesting cycles (~biweekly visits during nesting seasons and bimonthly visits during non‐nesting seasons). The nests were checked using a small mirror (8 × 5 cm) attached to a pole, and the stages of development were recorded for each chick or egg during each visit. Nests containing either an egg or chick at any point during this period were considered active.

Chick developmental trajectories were divided into nine different stages, modified from Lim and Earl of Cranbrook (2014), and included characteristic periods such as the egg stage, the newly hatched stage (chick without pin feathers), and the stage when pin feathers appear on the body (see Table A1 for a complete list of stages). The time taken by every chick in each stage was documented and averaged by species (Table A1). Our survey data were used to generate a time series of chick development. As our colony visits to monitor nesting were very frequent, there were only short gaps between survey days and we were able to interpolate the chick development stage using data between consecutive surveys, which were not more than a few days apart. We collapsed the nine developmental stages into three main phases: an egg phase (stage 0), a pin‐feather growth phase (development from featherless to fully feathered chick; stages 1–5 in Table A1) and a wing‐growth phase (characterized by extension of flight feathers or primaries on the wing; stages 6–8 in Table A1). The duration of each phase was calculated per species and compared using a Student's *t*‐test. In addition, the breeding activity of each species in the colony over the year was visualized by plotting the proportion of active nests (i.e., nests from that species that were active at some point over the course of the year) without offspring, or that had a developing swiftlet in one of the three major developmental phases (egg phase, pin‐growth phase, wing‐growth phase). The number of eggs present in the colony by species over the year was also plotted using a general additive model implemented in the program mgcv 1.8‐41 in R 3.5.2 (Wood, 2004; Wood, 2011), with observation date being subject to a smoothing parameter. The breeding period of each swiftlet species was compared with that of the resident avifauna of Singapore using the function “lm” from the R package lme4 v1.1.27.1 (Bates et al., 2015).

To investigate whether light levels or distance from the nearest entrance affected the nest distribution of each species differently, we collected light measurements at 3 m intervals along the bunker corridors using a mobile phone application (Lux Light Meter). Light measurements were collected at the same time on two separate days with similar weather conditions and then averaged. To obtain estimates of light values for each nest, LOESS curves were calculated for each corridor using the function “loess” in the R package “stats” (with light measurements plotted as a function of distance), and we extrapolated a light value for each individual nest based on its position along the corridor. For nests along short corridors with two light measurements or fewer, a LOESS curve could not be calculated, so we assigned the light value of the closest light readings to these nests (three nests in total). We then used Student's t‐tests to assess whether light exposure or distance from the nearest entrance differentially affected species‐specific nest distributions.

We assessed whether clustering of nests within the colony by species was greater than expected by chance. To do this, we ran a simulation which randomly shuffled swiftlet species identities (141 black‐nest swiftlets and 114 edible‐nest swiftlets) across all active nests (*n* = 255), and compared the simulation results (*n* = 10,000 iterations) with the species‐segregation data we observed within the colony.

### 2.4 Survival differences between species and across the colony

Using the nesting data collected during colony visits, we calculated survivorship at the individual level for each swiftlet egg and chick. Eggs were presumed to be dead if they did not hatch, disappeared from the nest, or had fallen out of the nest (the reason for these displacements remains unclear). Survival of swiftlet eggs and chicks was analyzed using the program survival 2.44 in R 3.5.2, which allows analysis of time‐related event data (Therneau & Grambsch, 2000). The data were first filtered to exclude observations that did not start with an egg, as well as those that did not conclude with either fledging, chick death, or egg death, resulting in a dataset of 133 nests from black‐nest swiftlets (347 egg observations, 242 chick observations) and 112 nests from edible‐nest swiftlets (586 egg observations, 286 chick observations). Survival was modeled as a function of species with a Cox proportional hazards regression model to investigate the association between survivorship and predictor variables, using the “coxph” function. The proportional hazards assumption (the assumption that each individual has a similar egg death or chick death probability which is scalable by species) was tested with the “cox.zph” function, and Kaplan–Meier survival curves were generated using the “survfit” function. A nonparametric log rank test (with the function “survdiff”) was used to test if survivorship between species differed significantly.

To investigate whether factors other than species identity influenced hatching and fledging success, we constructed two different linear mixed effects models using the function “lmer” from the R package lme4 v1.1.27.1 (Bates et al., 2015; Knief & Forstmeier, 2021). For this analysis, we used the reduced dataset described earlier. In the first model, egg‐hatching success was the dependent variable, distance from entrance and light values were designated as continuous covariates, species assignment was set as a fixed effect, (*n* = 2 levels) and nest identity (*n* = 245) and wall identity (*n* = 21) were specified as random effects to control for repeated measures. In the second model, for eggs that successfully hatched, chick‐fledging success was calculated using the same continuous covariates, fixed effects, and random effects as the first model. As the light values did not explain any of the variation seen in the model, we reran the two described models excluding light as a continuous covariate. We also ran additional versions of the two models in which we demarcated the “off‐season” breeding peak during which edible‐nest swiftlets showed breeding activity but black‐nest swiftlets showed none (Section 3), in order to compare variation in egg‐hatching and chick‐fledging success rates during this off‐season with rates measured across the rest of the year for edible‐nest swiftlets.

As both egg‐hatching and chick‐fledging success appeared to be significantly influenced by wall identities (Section 3), we additionally estimated offspring survival rates for each species and for each wall. First, we ran the two mixed effects models described above while removing the intercept (Bates et al., 2015; Pei et al., 2020) and fitting wall identities as a fixed effect (*n* = 16 levels) instead of a random effect, to study the walls' effect on offspring survival rates. For this analysis, we focused on walls with a high proportion of nesting activity (walls with <20 eggs across the entire monitoring period from both species combined were lumped in the category “rest”). We constructed four mixed effects models for pairwise combinations of egg hatching and chick fledging for each species independently, in order to visualize wall effects. We assumed Gaussian errors for all models (Schielzeth et al., 2020), and all dependent variables and covariates were Z‐scaled to compare the estimated standardized effect sizes of different factors from different models (Lakens, 2013; Knief & Forstmeier, 2021; Nakagawa & Cuthill, 2007; Nakagawa & Schielzeth, 2013).

Finally, to determine which walls were most successful in terms of relative fledging output, we focused on walls with a minimum of three active nests (for each species), and ranked the top five walls for each species as those that yielded the highest relative total fledging rates. To do this, we quantified nesting success using the metric of total fledging rate, that is, what percentage of eggs laid on that wall resulted in successful fledglings. In order to determine whether walls with the highest total fledging rates were beneficial for both species, we also assessed the extent to which walls with high total fledging rates for one species were—at the same time—highly ranked for the other species.

## 3 RESULTS

### 3.1 Echolocation call parameters

One of the most striking differences between the echolocation calls of the two species is that those of the black‐nest swiftlet contain a series of motifs of either single or double‐clicks, whereas those of the edible‐nest swiftlet contain only double‐clicks (Figure 1a, Figure A1c). The first sub‐click of the edible‐nest swiftlet is softer and has a lower maximum frequency than the second sub‐click, whereas the first sub‐click of the black‐nest swiftlet is significantly louder and of a higher maximum frequency than the second sub‐click (Figure 1, Table 1). We were unable to discern any difference in the context in which black‐nest swiftlets used single versus double‐click calls.

**TABLE 1:** Means and standard deviations (SD) of the vocal parameters for black‐nest and edible‐nest swiftlets that were diagnosable according to the Isler criterion: difference in maximum frequency between first and second sub‐clicks (*f*_max1_–*f*_max2_), average number of sub‐clicks per motif, ratio of maximum amplitude of the first element compared to the second (MA_1_/MA_2_), and ratio of peak amplitude of the first element compared to the second (PA_1_/PA_2_).

	*f*_max1_–*f*_max2_	No. of sub‐clicks	MA_1_/MA_2_	PA_1_/PA_2_
Black‐nest swiftlet mean	2984.99	1.48	2.37	2.36
Black‐nest swiftlet SD	2379.69	0.26	0.95	0.92
Edible‐nest swiftlet mean	−4575.28	2	0.36	0.36
Edible‐nest swiftlet SD	1748.34	0	0.14	0.13

Analysis of all the bioacoustic parameters associated with double‐click calls also revealed two distinct clusters on the PCA, which corresponded to the two species (Figure 1b). PC1 accounted for 50.24% of total variance and was positively correlated with minimum frequency of motifs, duration of interval between sub‐clicks, and maximum and minimum frequencies within the recording. PC2, accounting for 14.42% of total variance, was positively correlated with peak and center frequency, difference in maximum frequency between the first and second sub‐clicks, and the duration of the first sub‐click. In addition, 4 out of the 16 vocal parameters exhibited diagnosable differences between species using the Isler criterion: (1) difference in maximum frequency between the first and second sub‐click, (2) average number of sub‐clicks per motif, (3) ratio of maximum amplitude of the first sub‐click compared to the second, (4) ratio of peak amplitude of the first sub‐click compared to the second (Table 1).

### 3.2 Nesting ecology and overall differences in offspring survivorship

**FIGURE A2:**
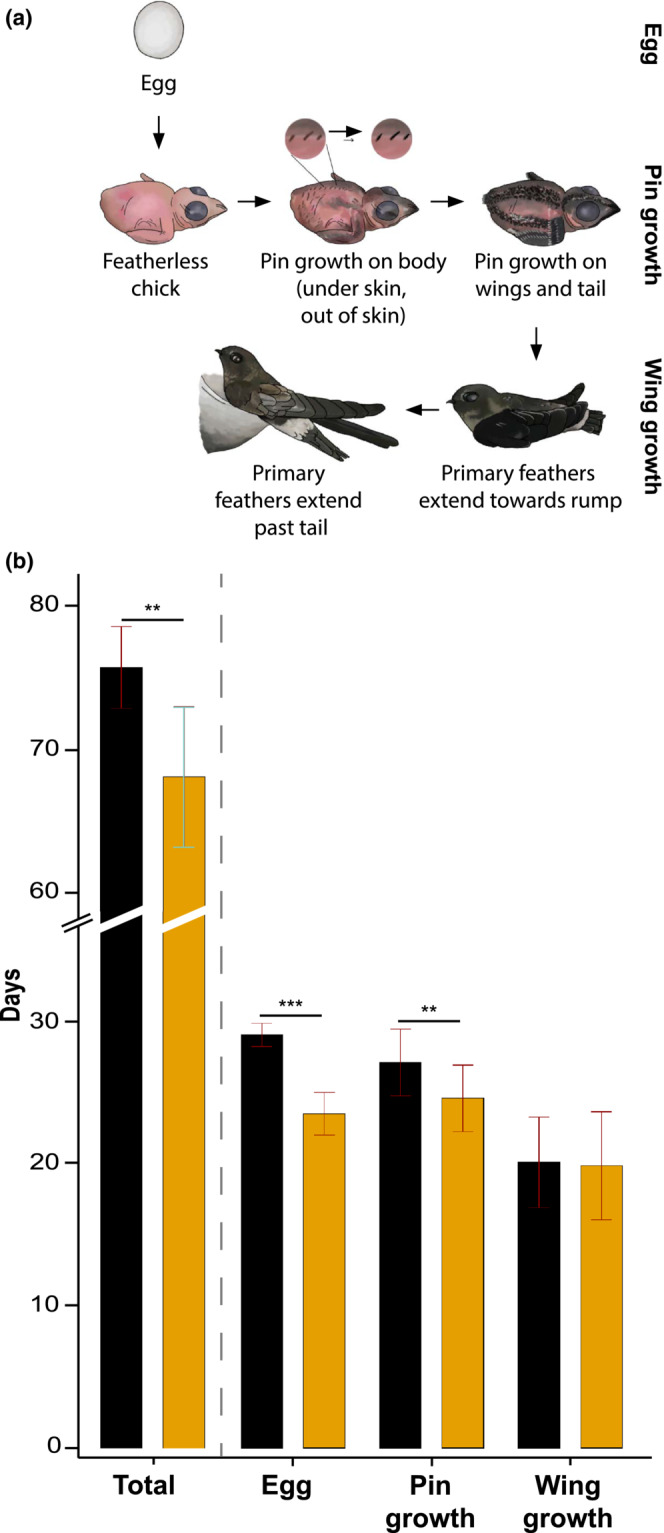
Longer developmental times in black‐nest compared to edible‐nest swiftlets. (a) Depictions of seven of the eight developmental stages of a typical swiftlet, from egg to fledgling. Major phases are labelled on the right of the illustrations. (b) A comparison of the average total development time (left) of black‐nest swiftlets (black) and edible‐nest swiftlets (orange) in days, and a breakdown of the average times taken for the three major phases (the egg phase [*n* = 66], the pin‐growth phase [*n* = 85], and the wing‐growth phase [*n* = 85]) (significance levels assessed using Student *t*‐test). Drawings by Hui Zhen Tan.

We estimate the population size of black‐nest swiftlets and edible‐nest swiftlets at the colony to be 302 and 228 adults, respectively. A total of 151 black‐nest swiftlet nests and 114 edible‐nest swiftlet nests showed activity during the monitoring period between August 2018 and August 2019. Black‐nest swiftlets invariably lay one egg per nest, whereas edible‐nest swiftlets lay two, and both parents participate in incubation and chick provisioning (Kang et al., 1991; Lim & Earl of Cranbrook, 2014). Egg incubation and chick‐brooding durations were on average longer for black‐nest swiftlets than for edible‐nest swiftlets (${\text{mean}}_{\mathrm{BNS}}$ = 29.5 days [SD = 0.5] versus ${\text{mean}}_{\mathrm{ENS}}$ = 23.4 days [SD = 1.54] and ${\text{mean}}_{\mathrm{BNS}}$ = 47.13 days [SD = 3.24] vs. ${\text{mean}}_{\mathrm{ENS}}$ = 44.43 days [SD = 4.62] respectively; Figure A2). We followed the trajectories of 347 black‐nest and 586 edible‐nest swiftlet eggs in full: 214 black‐nest swiftlets (61.6% of all egg observations) and 211 edible‐nest swiftlets (36% of egg observations) successfully developed until fledging.

**FIGURE 2:**
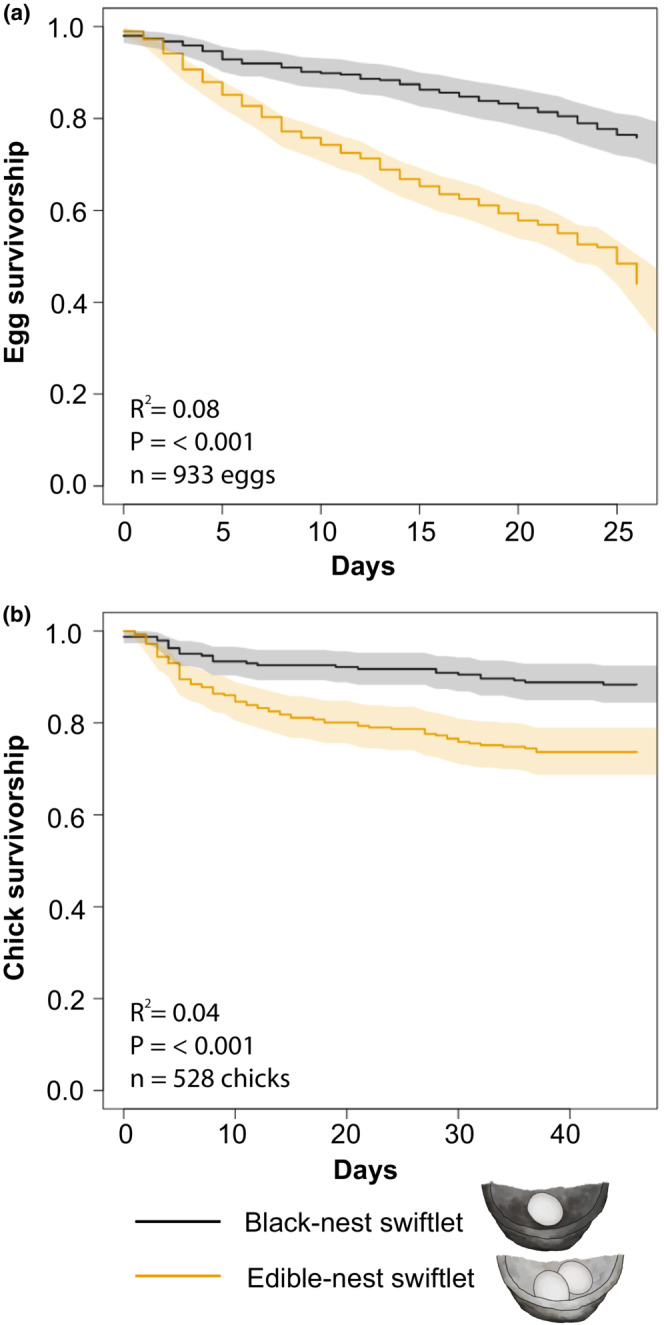
Higher offspring survivorship of black‐nest swiftlets (black line) compared to edible‐nest swiftlets (orange line). (a) Kaplan–Meier curve depicting the survivorship of eggs with 95% confidence intervals (CI) of 347 black‐nest swiftlets and 586 edible‐nest swiftlets. (b) Kaplan–Meier curve depicting the survivorship of chicks with 95% CIs of 242 black‐nest swiftlets and 286 edible‐nest swiftlets.

The offspring survival analyses were divided into two categories: the period from when the egg was first laid until it hatched, and the period from when the egg hatched until the chick fledged (Figure 2). Most reproductive failures are from the loss of an egg from the nest (for instance because it dropped to the ground or disappeared), or are from chick death prior to fledging. Statistics for egg survivorship (based on 347 black‐nest and 586 edible‐nest swiftlet eggs) and chick survivorship (based on 242 black‐nest and 286 edible‐nest swiftlet chicks) were calculated for the entire developmental period (in Figure 2, estimates until 26 days for eggs and 46 days for chicks are shown, as these values represent the average hatching and fledging times). The chick and egg datasets for both swiftlets passed the proportional hazards assumption test; the Cox regression model for egg survival had a higher *R* ^2^ value (.08) than the model for chick survival (.04), with both explaining a relatively low amount of the variance. Egg and chick survivorship was significantly higher in black‐nest than in edible‐nest swiftlets (Figure 2).

**FIGURE 3:**
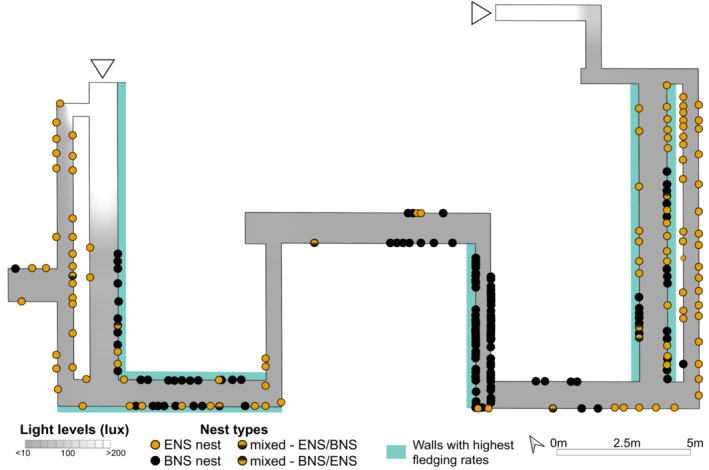
More black‐nest swiftlets nest on walls with the highest fledging rates. Map showing the corridor layout of the underground bunker used by swiftlets for colonial nesting in Sentosa, Singapore (for more details, see Figure A5). Corridors are shaded from grey to white based on the average light intensity. White triangles indicate the two entrances to the bunker (through which light enters). Each circle is a unique nest; the locations of the four different nest types (black‐nest swiftlet (BNS) nests, edible‐nest swiftlet (ENS) nests, and two mixed nest types) along corridor walls are depicted. Walls highlighted in teal have on average higher total chick fledging success (for black‐nest swiftlets and for edible‐nest swiftlets) than other walls (see Figure A5 for a breakdown by species); these walls have more black‐nest swiftlet nests (64 nests, or 48% of all BNS nests) than edible‐nest swiftlet nests (38 nests, or 34% of all ENS nests).

We obtained coordinates for each nest and mapped their spatial distribution within the colony (Figure 3). Active nests of four different types (white nests built by edible‐nest swiftlets, black nests built by black‐nest swiftlets, and two types of mixed nests) were identified along 21 of the total 28 walls of the bunker. The nests of edible‐nest swiftlets were found almost exclusively on corridors at the extreme ends closer to the entrances of the bunker, whereas the nests of most black‐nest swiftlets were found in corridors farthest from either entrance (Figure 3). We also observed species‐specific clustering of nests in the colony. Of the 21 walls with active nests, 13 walls were exclusively occupied by a single species, which is significantly greater than expected by random chance (none of our 10,000 simulations of shuffling species identity among available nests generated this result).

We examined whether distance from entrances or light levels affected the distribution of nests. On average, black‐nest swiftlets nest significantly farther from an entrance (deeper within the bunker) than do edible‐nest swiftlets (*p* < .001). However, nests of the two species were not exposed to significantly different amounts of light (*p* = .06), as both species appear to avoid bright locations—no active nests were recorded above a light reading of 60 lux, and over 99% of active nests were exposed to <10 lux (total darkness).

### 3.3 Developmental differences and factors influencing hatching and fledging success

Black‐nest swiftlets take more time (a week) to fledge than do edible‐nest swiftlets, and also spend significantly longer in both the egg and the pin‐feather phases (Figure A2). Analysis of hatching and fledging success rate of both species confirmed that the eggs of edible‐nest swiftlets exhibited significantly lower hatching success compared to those of black‐nest swiftlets (*b* = −.38, SE = 0.12, *p* < .01; Table A2). Unexpectedly, eggs from nests located further away from entrances were significantly less likely to hatch (*r* = −.22, SE = 0.08, *p* < .01), but the distance of the nest to the entrance did not influence fledging success (*r* = −.11, SE = 0.10, *p* = .30; Table A2).

**FIGURE A3:**
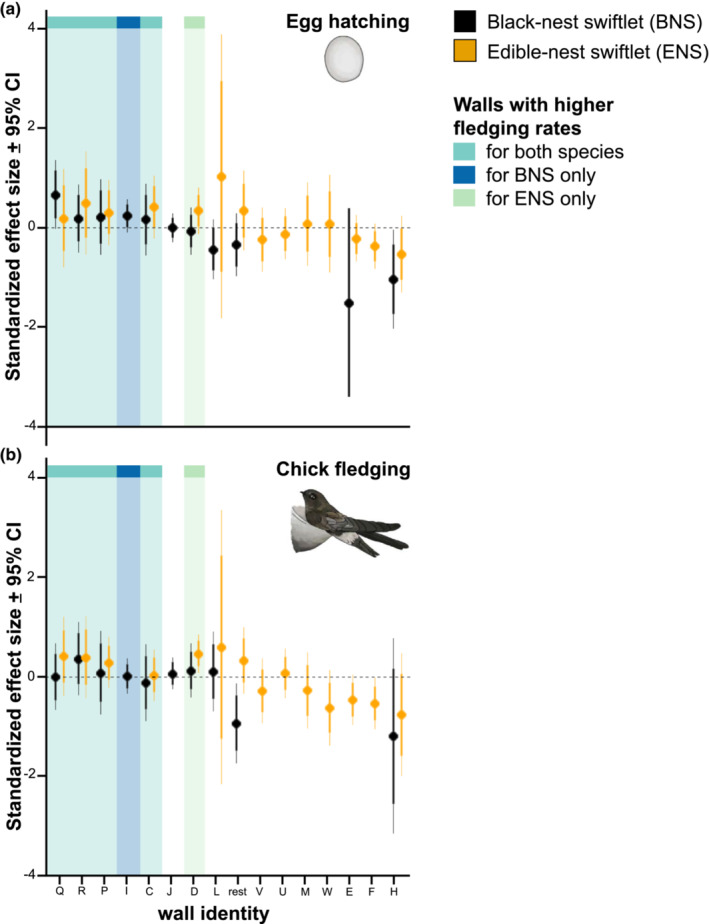
Standardized wall effect sizes with respective 95% confidence intervals (CIs) for egg‐hatching success (a) and chick‐fledging success (b), as estimated by the mixed effects model. Walls with the highest total fledging rates (for each species) are highlighted. Walls with a sample size lower than 20 eggs are combined in the category ‘rest’. Drawings by Hui Zhen Tan.

Nest identity appeared to significantly influence egg‐hatching success (*R*_nest_ = 16%, *p* < .0001); however, nest identity explained much less variation in fledging success (*R*_nest_ = 7%, *p* = .10). Interestingly, we found that wall identity significantly influenced the success of both hatching and fledging (for hatching success *R*_wal_ = 10%, *p* = .001; for fledging success *R*_wal_ = 19%, *p* < .0001; Table A2, see also Table S1 for effects on clutch success); therefore, we summarized the model's effects of each wall for each species (Table S2; Figure A3).

**FIGURE A4:**
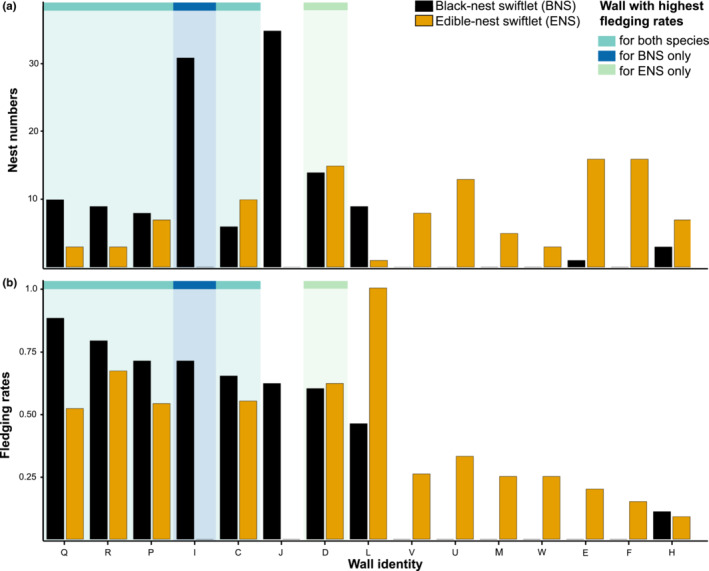
Number of nests and rates of fledging per wall, divided by species. (a) number and distribution of nests of black‐nest and edible‐nest swiftlets (black and orange, respectively) on the different walls in the bunker. (b) total fledging rates of black‐nest and edible‐nest swiftlets on the different walls in the bunker. Walls with the highest total fledging rates are highlighted. Nests of black‐nest swiftlets are more numerous but are distributed across fewer walls compared to those of edible‐nest swiftlets.

**FIGURE A5:**
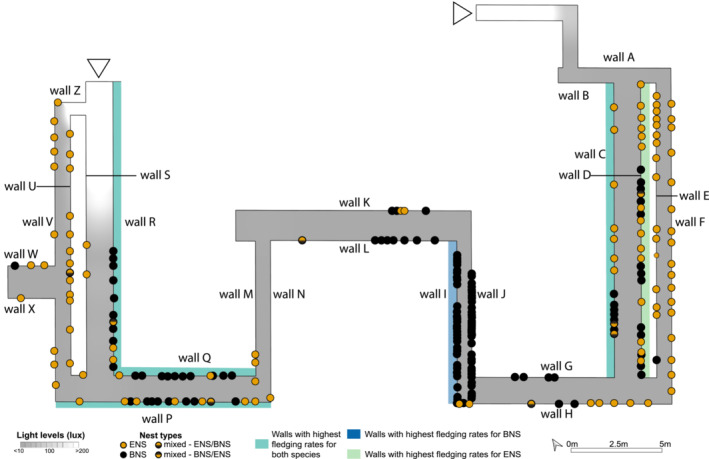
Map of bunker showing the walls with the highest fledging rates. Wall identifiers are indicated; walls with the highest fledging rates for black‐nest swiftlet (BNS) and edible‐nest swiftlet (ENS) are highlighted in blue and green, respectively, and walls that had the highest fledging rates for both species are highlighted in teal. Location of different nest types (BNS nests (black), ENS nests (orange), and two mixed nest types) along corridor walls are shown. Corridors are shaded from grey to white based on the average light intensity. White triangles indicate the two entrances to the bunker through which light enters.

To understand how wall identity impacts survival and to investigate whether some locations had higher overall fledging success (and perhaps represented prime nesting locations in the colony), we next examined the walls with the highest total fledging success (‘best walls’) for each species, and ranked the five best walls per species that had a minimum of three active nests (Figure A4b). For black‐nest swiftlets, the best walls were Q, R, P, I, and C (listed in order of total fledging success); and for edible‐nest swiftlets, R, D, C, P, and Q (Figures A4 and A5, Table S2). Interestingly, a higher number (and percentage) of black‐nest swiftlet nests compared to edible‐nest swiftlet nests were located on the species' respective best walls. Specifically, 64 of 133 black‐nest swiftlet nests were located on the five best walls for this species (48.1%), compared with 38 of 112 edible‐nest swiftlet nests (33.9%; Figure 3). Four of the five best walls were shared between the two species (Q, R, C, and P), suggesting that some aspect of these walls' location (or composition, see Section 4) was beneficial to the offspring survival for both species. We next looked at these shared walls to assess potential interspecific competition for nesting spots. Here, black‐nest swiftlet nests were also the most common: 33 were observed on these walls (24.8% of all black‐swiftlet nests) compared with 23 nests of edible‐nest swiftlet (20.5%).

### 3.4 Temporal differences in breeding

**FIGURE 4:**
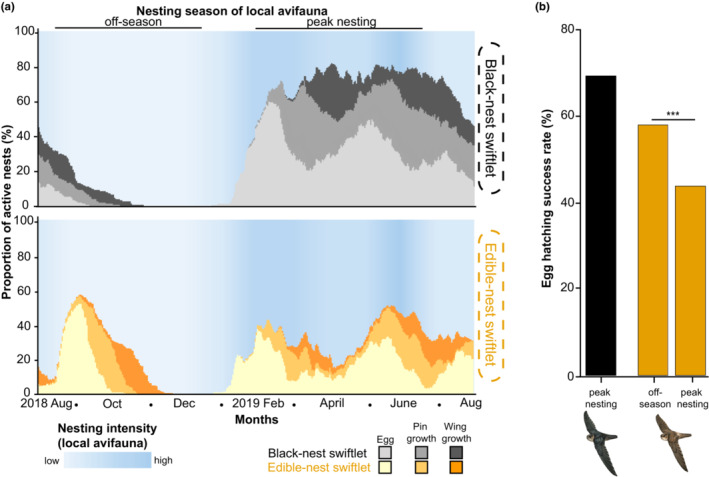
Edible‐nest swiftlets show higher egg‐hatching rates during off‐season breeding compared to peak season breeding. (a) Breeding phenology of black‐nest swiftlets (top) and edible‐nest swiftlets (bottom). Proportion of active nests (calculated from the total number of nests for each species in which breeding was attempted during the year of monitoring) indicated by cumulative curve height; nests with chicks of different developmental stages are indicated by shades of grey and yellow. As an example, when monitoring began in August 2018, 45% of nests of the black‐nest swiftlet had breeding activity (total height of grey areas), with 15% containing eggs (light grey), 20% containing chicks in the pin‐growth phase (grey), and 10% containing chicks in the wing‐growth phase (dark grey). See Figure A2 for more details on each developmental phase. Nesting intensity of avifauna in Singapore (as defined by Berman et al., 2022) is highest from February–June (indicated by darker blue) and lowest in September–December. The breeding peaks for both swiftlets are largely synchronous and coincide with the local peak nesting season, with the exception of an off‐season peak during September–November 2018, where an increase in breeding was observed only in edible‐nest swiftlets. (b) Edible‐nest swiftlet eggs (orange) laid during the off‐season breeding period have higher egg‐hatching success than eggs laid during peak season breeding periods (black‐nest swiftlet hatching success (black) shown for reference). Swiftlet illustrations are reproduced with permission of Lynx Edicions.

**FIGURE A6:**
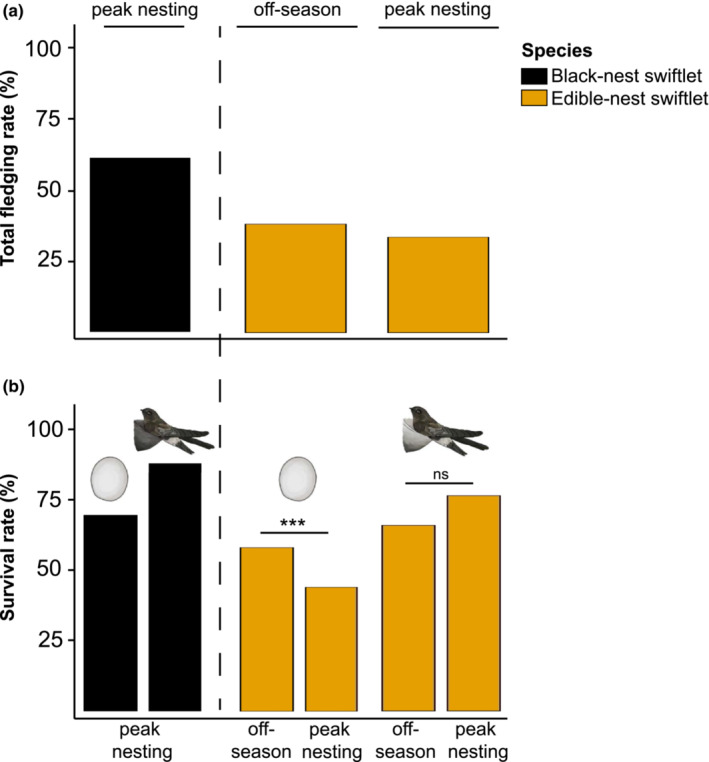
Rates of successful egg hatching of edible‐nest swiftlets higher during the off‐season than during the peak season. (a) Total fledging rates of black‐nest swiftlets during the peak season and edible‐nest swiftlets during off‐season and peak season. (b) Rates of successful egg hatching and chick fledging in black‐nest swiftlets during peak season, and rates of successful egg‐hatching and chick‐fledging compared between off‐season and peak season for edible‐nest swiftlets (*p* < .0001 for egg hatching, not significant (ns) for chick fledging; significance levels assessed using our mixed effects model).

During the year‐long monitoring, we observed multiple breeding peaks for both species, which is not unusual for birds breeding in the tropics (Berman et al., 2022). We compared the breeding peaks we observed in the swiftlets with nesting data from 56 other local bird species in Singapore (Berman et al., 2022). Interestingly, the black‐nest swiftlet displayed three overlapping breeding peaks, which corresponded significantly with the local avifaunal nesting season (*r* = .77, SE = 0.19, *p* = .003), and decreased in magnitude over the year (between February and August; Figure 4, Figure S1). In contrast, edible‐nest swiftlet breeding peaks showed poor correspondence with local avifauna (*r* = .41, SE = 0.29, *p* = .19; see also Figure S1). Although edible‐nest swiftlets also had three peaks (between January and August), they displayed an additional unique breeding peak between September and November, which fell within the local avifaunal off‐season (Figure 4; Figure S1). Unlike the three breeding peaks which were mostly synchronous between both swiftlets and other local avifauna, the additional fourth off‐season breeding period of edible‐nest swiftlets—characterized by the highest proportion of active nests—occurred at a time when black‐nest swiftlets did not breed (Figure 4; Figure S1). This additional off‐season peak by edible‐nest swiftlets exhibited significantly higher egg‐hatching success than the three edible‐nest breeding peaks between January and August (mean difference of 14%, *r* = .34, SE = 0.08, *p* < .0001; Figure 4b, Figure A6).

## 4 DISCUSSION

Resource partitioning has been commonly documented in animals which breed at higher densities within a limited area, such as colony nesters (Bretagnolle et al., 1990; Brown & Brown, 2001; Burger & Shisler, 1978; Hunter, 1983; Monteiro & Furness, 1998; Navarro et al., 2013; Oro et al., 2009). In our study, we surveyed two congeneric echolocating swiftlets, both of which nest colonially in an abandoned World War II bunker, to determine how they differ, and whether they partition resources spatially and/or temporally. We found significant differences in the types of echolocation calls between the two species: whereas edible‐nest swiftlets produce only double‐click calls, black‐nest swiftlets emit a mix of both double‐ and single‐click calls. We also observed differences in egg numbers, developmental times, and offspring survivorship between the two species and demonstrate interspecific spatial and temporal segregation in the colony.

### 4.1 Differences in echolocation calls

The echolocating calls of *Aerodramus* swiftlets are produced in the form of clicks, which are audible to the human ear (Thomassen et al., 2003), and are used exclusively for navigation. The double‐click call comprises two sub‐clicks in quick succession, and is more widespread across the genus than the single‐click call, which is assumed to be the more derived type of call and is known only from two swiftlets (black‐nest swiftlet and Atiu swiftlet, *A. sawtelli*) (Fullard et al., 2010; Price et al., 2004). Here, we demonstrate that black‐nest swiftlets, the only species with single‐ and double‐click calls, produce both calls at roughly equal frequency (mean number of clicks = 1.48) to navigate in the dark (Table 1).

Vocalizations that are known to play an important role in avian sexual selection (such as song) often differ between closely related species. By contrast, vocalizations that serve a sensory purpose, such as calls used in navigation, are expected to be highly similar between species, as they are likely governed by functional constraints (rather than sexual selection) (Bradbury & Vehrencamp, 1998; Cibois et al., 2018). These functional constraints may lead to differences in pitch or frequency of calls (Demery et al., 2021). We observe differences in frequency, amplitude, and also in call structure between the two swiftlets; the latter is not expected to result from body size differences alone. This suggests that the swiftlets' call differences may serve a specific purpose such as interspecific communication, as has been shown in other echolocating taxa, such as bats and porpoises (Fenton et al., 2016; Jones, 2008). In addition, it is possible that differences between the echolocation calls of black‐ and edible‐nest swiftlets may have resulted from character displacement (Thomassen, 2005), facilitating the recognition of conspecifics within the colony (Medway, 1962a; Price et al., 2004; Thomassen et al., 2004).

### 4.2 Interspecific spatial segregation within the colony

Competition between ecologically similar species in a community may be reduced by spatial segregation, enabling different parts of a habitat to be used by different species (Granroth‐Wilding & Phillips, 2019; Schoener, 1974). Mixed colonies of black‐nest and edible‐nest swiftlets have previously been shown to display interspecific spatial segregation in nesting. In populations of natural cave‐nesting swiftlets in Borneo, each species tends to congregate in separate groups.

Despite the limited available nesting area at our study site, we clearly observe strong interspecific nest segregation: 13 of the 21 walls with active nests were exclusively occupied by a single species (Figure 3), which is significantly greater than expected by chance. The lack of complete interspecific segregation, such as those observed in Bornean caves, and the presence of mixed nests at our study site, may be due to limited acceptable nesting space within the Sentosa colony. This colony is situated in an underground bunker with walls that are under 3 m tall, leaving little space for clusters of conspecific nests to form; nest clusters are spread laterally, unlike those in caves, which can reach over a 100 m in height (Lim & Earl of Cranbrook, 2014).

Some studies on colonially nesting birds have observed higher mortality rates among chicks at colony edges than at the center (Coulson, 1968), suggesting that colony interiors are favored. Interestingly, our data did not support this pattern, as our model showed significantly reduced hatching success at the colony center (there was however no influence on chick fledging rates; Table A2).

Our analysis of offspring survival rates revealed that nest location within the colony—in particular, which specific wall the nest was located on (Figure 3 and Figure A3, Tables A2, Tables S1 and S2)—was a significant predictor of both egg‐hatching and chick‐fledging success. Additionally, when the five best walls for each species were ranked independently, we saw large overlap in the best walls for each species (four of the five best walls for each species were shared between species; Figures S3 and S5), implying that both species may have similar nesting preferences within this colony.

In line with expectations from other studies documenting spatial segregation at breeding sites between ecologically similar species (Burger & Shisler, 1978; Shelley et al., 2004; Zeng & Lu, 2009), the larger black‐nest swiftlet has higher representation on the best walls (Figure 3). That some nests on specific walls had higher hatching and fledging rates may be due to structural or surface features of the wall (such as moisture levels) that could affect adhesion or nest properties; understanding the determinants of wall quality will require future studies at this site. Based on our current data, we are unsure what features of the walls specifically influence nest success or failure.

### 4.3 Temporal shifts in breeding seasons and differences in offspring survival

Swiftlets at our site have a protracted breeding season (a feature also observed in cave‐roosting populations; Medway, 1962b), comprised of multiple peaks that extend across most of the year; edible‐nest swiftlets appear to have a longer breeding season and more breeding peaks compared to black‐nest swiftlets (Figure 4). Both swiftlet species breed during largely similar periods (except for the edible‐nest swiftlets' off‐season peak), with black‐nest swiftlets showing significant correlation with the annual nesting trends of the local avifauna (Figure 4; Berman et al., 2022).

Breeding seasonality in the equatorial tropics appears to be correlated with the number of hours per day of sunshine (during which direct irradiance exceeds 120 Watts/m^2^). In Singapore, daily hours of sunshine are highly influenced by rainfall and cloud cover (Berman et al., 2022; Medway & Wells, 1976). Periods of high sunshine probably especially benefit aerial insectivores like swiftlets, allowing them to spend more time foraging in flight to meet the increased food demand required for chick provisioning. In regions where bird breeding activity is confined to a few months of the year, the asynchrony in breeding times that has been observed in colonial nesting species is thought to reduce interspecific competition (Burger & Shisler, 1978; Hunter, 1983; Monteiro & Furness, 1998; Navarro et al., 2013). When shifts in breeding times occur, the off‐set is typically short (on the scale of a few weeks, as in giant petrels and prions) to allow chick rearing during periods when food is most abundant (Jones et al., 2020; Monteiro & Furness, 1998; Navarro et al., 2013). Interestingly, we observed an additional nesting peak by edible‐nest swiftlets from September to November, during which black‐nest swiftlets do not appear to be actively laying eggs (but still utilize the bunker for roosting). As overall avifaunal nesting activity across Singapore is also low during this time (Figure 4), this period may represent a sub‐optimal time for local nesting. Yet, surprisingly, edible‐nest swiftlets appear to lay more eggs and display significantly higher rates of egg‐hatching during this off‐season peak than during the peak season (Figure 4). Our results suggest that edible‐nest swiftlets benefit from nesting at a time when black‐nest swiftlets are not. These findings are in line with other work which has shown that smaller species are more likely to shift temporal behaviors than are larger species (Pei et al., 2018).

## 5 CONCLUSIONS

Our study finds evidence of spatial segregation in nesting patterns, and staggered breeding seasonality between two species sharing a nesting site. Edible‐nest swiftlets benefit from breeding at a time when black‐nest swiftlets are not concurrently nesting in the colony, displaying higher reproductive output during this off‐season compared to the peak season when both species are nesting. Additionally, nesting space on walls with high fledging rates is preferentially claimed by larger black‐nest swiftlets. The swiftlets also differ behaviorally, exhibiting significant differences in their echolocation call structure despite their morphological similarity. Future studies into the microclimate within this colony may reveal why some locations have higher hatching and fledging success than others, and examination of dietary differences between the species (and also across seasons) may determine to what extent other resources such as food are also partitioned. Our study sheds light on how ecologically similar species may partition multiple resources within a confined environment.

## AUTHOR CONTRIBUTIONS

**Keren R. Sadanandan:** Conceptualization (equal); data curation (lead); formal analysis (lead); investigation (lead); methodology (lead); validation (lead); visualization (lead); writing – original draft (equal); writing – review and editing (equal). **Hui Zhen Tan:** Conceptualization (equal); data curation (lead); formal analysis (lead); investigation (lead); methodology (lead); visualization (lead); writing – original draft (lead). **Hong Yao Lim:** Conceptualization (lead); data curation (lead); formal analysis (lead); investigation (lead); methodology (lead); writing – original draft (lead). **Yi Gain Tan:** Formal analysis (supporting); methodology (supporting); validation (supporting); visualization (supporting); writing – review and editing (supporting). **Grace Lee:** Project administration (supporting); resources (supporting); writing – review and editing (supporting). **Lena Chan:** Conceptualization (supporting); project administration (supporting); writing – review and editing (supporting). **Yifan Pei:** Formal analysis (equal); visualization (equal); writing – original draft (equal); writing – review and editing (supporting). **Frank E. Rheindt:** Conceptualization (lead); funding acquisition (lead); resources (equal); supervision (lead); writing – original draft (equal); writing – review and editing (equal). **Maude W. Baldwin:** Conceptualization (equal); funding acquisition (equal); resources (equal); supervision (lead); writing – original draft (lead); writing – review and editing (lead).

## CONFLICT OF INTEREST STATEMENT

The authors declare no conflict of interest.

## Supporting information


Appendix S1.

## Data Availability

Supporting data, model structures, and R scripts have been deposited in Dryad: https://doi.org/10.5061/dryad.wstqjq2px.

## APPENDIX 1

**TABLE A1:** Mean time taken (in days) by black‐nest swiftlets and edible‐nest swiftlets in each of the developmental stages, and the corresponding phase to which each stage is linked.

Phase	Egg	Pin stages					Wing stages			Total
Stage	0	1	2	3	4	5	6	7	8	0–8
	Unhatched egg	Chick without pins	Pins grow under skin on body	Pins emerge from skin on body	Feathers on head and body, pins on wings and tail	Feathers on wings and tail	Primary feathers extend past rump band	Primary feathers grow past tail	Primary feathers grow past tail by over 2.5cm	Total develop‐mental time
Black‐nest swiftlet	29.00 ± 0.82	3.44 ± 0.89	5.00 ± 1.41	5.58 ± 1.22	5.80 ± 1.20	7.53 ± 2.61	7.31 ± 2.10	8.18 ± 2.17	3.12 ± 1.78	75.75 ± 2.87
Edible‐nest swiftlet	23.47 ± 1.50	3.39 ± 0.69	5.13 ± 0.98	6.65 ± 1.37	4.75 ± 1.51	4.62 ± 1.10	5.54 ± 1.85	10.24 ± 2.69	3.75 ± 2.23	68.06 ± 4.92

**TABLE A2:** Predictors of the egg‐hatching success and chick‐fledging success of edible‐nest swiftlets (ENS) and black‐nest swiftlets (BNS) within the colony.

Model	Predictor	Estimate	SE	Lower CI	Upper CI	*Z*‐value	*p*
Egg hatching *N*_egg_ = 933	Intercept	0.232	0.111	−0.03	0.494	2.09	
	Species (ENS)	−0.383	0.122	−0.669	−0.097	−3.15	.002
	Distance from nearest entrance	−0.219	0.077	−0.4	−0.037	−2.83	.006
	Var (nest identity)	0.155					<.0001
	Var (wall identity)	0.096					.0002
	Var (residual)	0.729					
Chick fledging *N*_chicks_ = 528	Intercept	−0.023	0.138	−0.348	0.302	−0.17	
	Species (ENS)	−0.198	0.135	−0.517	0.121	−1.47	.13
	Distance from nearest entrance	−0.105	0.1	−0.341	0.131	−1.05	.3
	Var (nest identity)	0.065					.1
	Var (wall identity)	0.191					<.0001
	Var (residual)	0.795					

*Note*: Species identity was listed as a fixed effect (2 levels, with BNS as the baseline). Estimates are shown with their upper and lower 95% confidence intervals (calculated using ‘glht’ function from the R package ‘multcomp v.1.4‐18’).

Abbreviations: CIs, confidence intervals; SE, standard error; var, variance.

## References

[ece39805-bib-0001] Andreas, M. , Reiter, A. , Cepáková, E. , & Uhrin, M. (2013). Body size as an important factor determining trophic niche partitioning in three syntopic rhinolophid bat species. Biologia, 68(1), 170–175.

[ece39805-bib-0002] Bates, D. , Mächler, M. , Bolker, B. , & Walker, S. (2015). Fitting linear mixed‐effects models using lme4. Journal of Statistical Software, 67(1), 1–48.

[ece39805-bib-0003] Berman, L. , Li, D. , Shufen, Y. , Kennewell, M. , & Rheindt, F. E. (2022). Bird breeding season linked to sunshine hours in a marginally seasonal equatorial climate. Journal of Ornithology, 164, 1–14.

[ece39805-bib-0004] Bradbury, J. , & Vehrencamp, S. (1998). Principles of animal communication. Sinauer Associates.

[ece39805-bib-0005] Bretagnolle, V. , Zotier, R. , & Jouventin, P. (1990). Comparative population biology of four prions (genus *Pachyptila*) from the Indian Ocean and consequences for their taxonomic status. The Auk, 107(2), 305–316.

[ece39805-bib-0006] Brinkløv, S. , Fenton, M. B. , & Ratcliffe, J. M. (2013). Echolocation in oilbirds and swiftlets. Frontiers in Physiology, 4, 123. 23755019 10.3389/fphys.2013.00123 PMC3664765

[ece39805-bib-0007] Brown, C. R. , & Brown, M. B. (2001). Avian coloniality. In Current ornithology (pp. 1–82). Springer.

[ece39805-bib-0008] Brown, R. M. , Techow, N. M. , Wood, A. G. , & Phillips, R. A. (2015). Hybridization and back–crossing in giant petrels (*Macronectes giganteus* and *M. halli*) at Bird Island, South Georgia, and a summary of hybridization in seabirds. PLoS One, 10, e0121688. 25815478 10.1371/journal.pone.0121688 PMC4376808

[ece39805-bib-0009] Burger, J. , & Shisler, J. (1978). Nest site selection and competitive interactions of herring and laughing gulls in New Jersey. The Auk, 95(2), 252–266.

[ece39805-bib-0010] Cibois, A. , Thibault, J. C. , McCormack, G. , & Pasquet, E. (2018). Phylogenetic relationships of the Eastern Polynesian swiftlets (*Aerodramus*, Apodidae) and considerations on other Western Pacific swiftlets. Emu–Austral Ornithology, 118(3), 247–257.

[ece39805-bib-0011] Coulson, J. C. (1968). Differences in the quality of birds nesting in the centre and on the edges of a colony. Nature, 217(5127), 478–479.

[ece39805-bib-0012] Cros, E. , & Rheindt, F. E. (2017). Massive bioacoustic analysis suggests introgression across Pleistocene land bridges in *Mixornis* tit‐babblers. Journal of Ornithology, 158(2), 407–419.

[ece39805-bib-0013] Demery, A.‐J. C. , Burns, K. J. , & Mason, N. A. (2021). Bill size, bill shape, and body size constrain bird song evolution on a macroevolutionary scale. The Auk, 138(2), ukab011.

[ece39805-bib-0014] Eaton, J. A. , van Balen, B. , Brickle, N. W. , & Rheindt, F. E. (2021). Birds of the Indonesian archipelago (Vol. 2). Lynx Edicions.

[ece39805-bib-0015] Fenton, M. B. , Grinnell, A. D. , Popper, A. N. , & Fay, R. R. (2016). Bat bioacoustics (Vol. 54). Springer.

[ece39805-bib-0016] Fullard, J. H. , Barclay, R. M. , & Thomas, D. W. (2010). Observations on the behavioural ecology of the Atiu Swiftlet *Aerodramus sawtelli* . Bird Conservation International, 20(4), 385–391.

[ece39805-bib-0017] Granroth‐Wilding, H. M. , & Phillips, R. A. (2019). Segregation in space and time explains the coexistence of two sympatric sub‐Antarctic petrels. Ibis, 161(1), 101–116.

[ece39805-bib-0018] Grant, B. R. , & Grant, P. R. (1979). Darwin's finches: Population variation and sympatric speciation. Proceedings of the National Academy of Sciences, 76(5), 2359–2363. 10.1073/pnas.76.5.2359 PMC383600 16592654

[ece39805-bib-0019] Gwee, C. Y. , Eaton, J. A. , Garg, K. M. , Alström, P. , Van Balen, S. , Hutchinson, R. O. , Prawiradilaga, D. M. , Le, M. H. , & Rheindt, F. E. (2019). Cryptic diversity in *Cyornis* (Aves: Muscicapidae) jungle‐flycatchers flagged by simple bioacoustic approaches. Zoological Journal of the Linnean Society, 186(3), 725–741.

[ece39805-bib-0020] Heller, K. G. , & Helversen, O. V. (1989). Resource partitioning of sonar frequency bands in rhinolophoid bats. Oecologia, 80(2), 178–186. 28313104 10.1007/BF00380148

[ece39805-bib-0021] Hunter, S. (1983). The food and feeding ecology of the giant petrels *Macronectes halli* and *M. giganteus* at South Georgia. Journal of Zoology, 200(4), 521–538.

[ece39805-bib-0022] Hutchinson, G. E. (1991). Population studies: Animal ecology and demography. Bulletin of Mathematical Biology, 53(1), 193–213.

[ece39805-bib-0023] Isler, M. L. , Isler, P. R. , & Whitney, B. M. (1998). Use of vocalizations to establish species limits in antbirds (Passeriformes: Thamnophilidae). The Auk, 115(3), 577–590.

[ece39805-bib-0024] Jones, C. W. , Phillips, R. A. , Grecian, W. J. , & Ryan, P. G. (2020). Ecological segregation of two superabundant, morphologically similar, sister seabird taxa breeding in sympatry. Marine Biology, 167(4), 1–16.

[ece39805-bib-0025] Jones, G. (2008). Sensory ecology: Echolocation calls are used for communication. Current Biology, 18(1), R34–R35. 18177712 10.1016/j.cub.2007.10.056

[ece39805-bib-0026] Kang, N. , Hails, C. J. , & Sigurdsson, J. B. (1991). Nest construction and egg‐laying in edible‐nest swiftlets *Aerodramus* spp. and the implications for harvesting. Ibis, 133(2), 170–177.

[ece39805-bib-0027] Knief, U. , & Forstmeier, W. (2021). Violating the normality assumption may be the lesser of two evils. Behavior Research Methods, 53(6), 2576–2590. 33963496 10.3758/s13428-021-01587-5 PMC8613103

[ece39805-bib-0028] Lakens, D. (2013). Calculating and reporting effect sizes to facilitate cumulative science: A practical primer for t‐tests and ANOVAs. Frontiers in Psychology, 4, 863. 24324449 10.3389/fpsyg.2013.00863 PMC3840331

[ece39805-bib-0029] Leonard, M. L. , & Fenton, M. B. (1984). Echolocation calls of *Euderma maculatum* (Vespertilionidae): Use in orientation and communication. Journal of Mammalogy, 65(1), 122–126.

[ece39805-bib-0030] Lim, C. K. , & Earl of Cranbrook . (2014). Swiftlets of Borneo: Builders of edible nests (2nd ed.). Natural History Publications.

[ece39805-bib-0031] Lim, K. S. (2009). The Avifauna of Singapore. Nature Society (Singapore), Bird Group Records Committee.

[ece39805-bib-0032] MacArthur, R. , & Levins, R. (1967). The limiting similarity, convergence, and divergence of coexisting species. The American Naturalist, 101(921), 377–385.

[ece39805-bib-0033] Marcone, M. F. (2005). Characterization of the edible bird's nest the “caviar of the east”. Food Research International, 38(10), 1125–1134.

[ece39805-bib-0034] Medway, L. (1962a). The swiftlets (*collocalia*) of Niah cave, Sarawak part 2. Ecology and the regulation of breeding. Ibis, 104(2), 228–245.

[ece39805-bib-0035] Medway, L. (1962b). The relation between the reproductive cycle, moult and changes in the sublingual salivary glands of the swiftlet *Collocalia maxima* . Proceedings of the Zoological Society of London, 138(2), 305–315.

[ece39805-bib-0036] Medway, L. , & Wells, D. R. (1976). The birds of the Malay peninsula (Vol. 5). Witherby.

[ece39805-bib-0037] Monteiro, L. R. , & Furness, R. W. (1998). Speciation through temporal segregation of Madeiran storm petrel (*Oceanodroma castro*) populations in the Azores? Philosophical Transactions of the Royal Society of London Series B: Biological Sciences, 353(1371), 945–953.

[ece39805-bib-0038] Nakagawa, S. , & Cuthill, I. C. (2007). Effect size, confidence interval and statistical significance: A practical guide for biologists. Biological Reviews, 82(4), 591–605. 17944619 10.1111/j.1469-185X.2007.00027.x

[ece39805-bib-0039] Nakagawa, S. , & Schielzeth, H. (2013). A general and simple method for obtaining R2 from generalized linear mixed‐effects models. Methods in Ecology and Evolution, 4(2), 133–142.

[ece39805-bib-0040] Navarro, J. , Votier, S. C. , Aguzzi, J. , Chiesa, J. J. , Forero, M. G. , & Phillips, R. A. (2013). Ecological segregation in space, time and trophic niche of sympatric planktivorous petrels. PLoS One, 8, e62897. 23646155 10.1371/journal.pone.0062897 PMC3639899

[ece39805-bib-0041] Nicholls, B. , & Racey, P. A. (2006). Habitat selection as a mechanism of resource partitioning in two cryptic bat species *Pipistrellus pipistrellus* and *Pipistrellus pygmaeus* . Ecography, 29(5), 697–708.

[ece39805-bib-0042] Norris, K. S. , Prescott, J. H. , Asa‐Dorian, P. V. , & Perkins, P. (1961). An experimental demonstration of echolocation behavior in the porpoise, *Tursiops truncatus* (Montagu). The Biological Bulletin, 120(2), 163–176.

[ece39805-bib-0043] Oro, D. , Pérez‐Rodríguez, A. , Martínez‐Vilalta, A. , Bertolero, A. , Vidal, F. , & Genovart, M. (2009). Interference competition in a threatened seabird community: A paradox for a successful conservation. Biological Conservation, 142(8), 1830–1835.

[ece39805-bib-0044] Pacala, S. , & Roughgarden, J. (1982). Resource partitioning and interspecific competition in two two–species insular *Anolis* lizard communities. Science, 217(4558), 444–446. 17782979 10.1126/science.217.4558.444

[ece39805-bib-0045] Pei, Y. , Forstmeier, W. , Wang, D. , Martin, K. , Rutkowska, J. , & Kempenaers, B. (2020). Proximate causes of infertility and embryo mortality in captive zebra finches. The American Naturalist, 196(5), 577–596. 10.1086/710956 33064590

[ece39805-bib-0046] Pei, Y. , Valcu, M. , & Kempenaers, B. (2018). Interference competition pressure predicts the number of avian predators that shifted their timing of activity. Proceedings of the Royal Society B, 285, 20180744. 29875306 10.1098/rspb.2018.0744 PMC6015849

[ece39805-bib-0047] Price, J. J. , Johnson, K. P. , & Clayton, D. H. (2004). The evolution of echolocation in swiftlets. Journal of Avian Biology, 35(2), 135–143.

[ece39805-bib-0048] QGIS Development Team . (2022). QGIS geographic information system. Open Source Geospatial Foundation Project. http://qgis.osgeo.org

[ece39805-bib-0049] R Core Team . (2021). R: A language and environment for statistical computing. Computer Software.

[ece39805-bib-0050] Rheindt, F. E. , Norman, J. A. , & Christidis, L. (2014). Extensive diversification across islands in the echolocating *Aerodramus* swiftlets. Raffles Bulletin of Zoology, 62, 89–99.

[ece39805-bib-0051] Rheindt, F. E. , Prawiradilaga, D. M. , Ashari, H. , Suparno , Gwee, C. Y. , Lee, G. W. , Wu, M. Y. , & Ng, N. S. (2020). A lost world in Wallacea: Description of a montane archipelagic avifauna. Science, 367(6474), 167–170. 31919216 10.1126/science.aax2146

[ece39805-bib-0052] Schielzeth, H. , Dingemanse, N. J. , Nakagawa, S. , Westneat, D. F. , Allegue, H. , Teplitsky, C. , Réale, D. , Dochtermann, N. A. , Garamszegi, L. Z. , & Araya‐Ajoy, Y. G. (2020). Robustness of linear mixed‐effects models to violations of distributional assumptions. Methods in Ecology and Evolution, 11(9), 1141–1152.

[ece39805-bib-0053] Schoener, T. W. (1974). Competition and the form of habitat shift. Theoretical Population Biology, 6(3), 265–307. 4460260 10.1016/0040-5809(74)90013-6

[ece39805-bib-0054] Shelley, E. L. , Tanaka, M. Y. , Ratnathicam, A. R. , & Blumstein, D. T. (2004). Can Lanchester's laws help explain interspecific dominance in birds? The Condor, 106(2), 395–400.

[ece39805-bib-0055] Sin, Y. C. K. , Eaton, J. A. , Hutchinson, R. O. , & Rheindt, F. E. (2022). Re‐assessing species limits in a morphologically cryptic Australasian kingfisher lineage (Coraciiformes: Halcyonidae) using bioacoustic data. Biological Journal of the Linnean Society, 136(4), 489–505.

[ece39805-bib-0056] Therneau, T. M. , & Grambsch, P. M. (2000). The cox model. In Modeling survival data: Extending the cox model (pp. 39–77). Springer.

[ece39805-bib-0057] Thomassen, H. A. (2005). Swift as sound. Design and evolution of the echolocation system in swiftlets (Apodidae: Collocaliini) (Doctoral dissertation). Leiden University.

[ece39805-bib-0058] Thomassen, H. A. , Djasim, U. M. , & Povel, G. D. E. (2004). Echoclick design in swiftlets: Single as well as double clicks. Ibis, 146(1), 173–174.

[ece39805-bib-0059] Thomassen, H. A. , Wiersema, A. T. , de Bakker, M. A. , de Knijff, P. , Hetebrij, E. , & Povel, G. D. E. (2003). A new phylogeny of swiftlets (Aves: Apodidae) based on cytochrome‐b DNA. Molecular Phylogenetics and Evolution, 29(1), 86–93. 12967609 10.1016/s1055-7903(03)00066-6

[ece39805-bib-0061] Wood, S. N. (2004). Stable and efficient multiple smoothing parameter estimation for generalized additive models. Journal of American Statistical Association, 111, 673–686.

[ece39805-bib-0062] Wood, S. N. (2011). Fast stable restricted maximum likelihood and marginal likelihood estimation of semiparametric generalized linear models. Journal of the Royal Statistical Society (B), 73(1), 3–36.

[ece39805-bib-0063] Zeng, X. , & Lu, X. (2009). Interspecific dominance and asymmetric competition with respect to nesting habitats between two snowfinch species in a high‐altitude extreme environment. Ecological Research, 24(3), 607–616.

