# Supplementary material for: Spatial and temporal resource partitioning in a mixed‐species colony of avian echolocators
Source: Ecol Evol. 2023 Feb 16;13(2):e9805. doi: 10.1002/ece3.9805 (PMC9936513; doi:10.1002/ece3.9805)
Supplement: Supplementary file 1 — Appendix S1. [file ECE3-13-e9805-s001.pdf]

# SUPPORTING INFORMATION

**Table S1.** Wall effects on egg hatching and chick-fledging successes of the two swiftlet species. Estimated standardized effect sizes of walls on egg-hatching and chick-fledging successes of the two swiftlet species are shown with their standard error (SE) and Z-values. The raw survival rates of edible-nest swiftlets (ENS) and black-nest swiftlets (BNS) for each wall are shown with their sample sizes. Walls are listed according to their position in the bunker (from left to right; Figure S5). Walls with a sample size lower than 20 eggs are combined in the category 'rest'.

| | wall ID | estimate | SE | Z-value | survival rate | | sample size | |
|---|---|---|---|---|---|---|---|---|
| | | | | | ENS | BNS | ENS | BNS |
| Egg hatching | W | 0.026 | 0.327 | 0.078 | 0.522 | – | 24 | – |
| | V | -0.276 | 0.238 | -1.158 | 0.365 | – | 43 | – |
| | U | -0.176 | 0.204 | -0.863 | 0.42 | – | 70 | – |
| | R | 0.441 | 0.205 | 2.157 | 0.732 | 0.781 | 15 | 18 |
| | P | 0.33 | 0.182 | 1.808 | 0.637 | 0.792 | 46 | 14 |
| | Q | 0.583 | 0.199 | 2.933 | 0.579 | 1 | 21 | 17 |
| | M | 0.021 | 0.292 | 0.072 | 0.52 | – | 25 | – |
| | L | -0.098 | 0.22 | -0.444 | 1 | 0.493 | 1 | 24 |
| | I | 0.488 | 0.124 | 3.928 | – | 0.802 | – | 78 |
| | J | 0.266 | 0.111 | 2.399 | – | 0.694 | – | 106 |
| | H | -0.625 | 0.225 | -2.777 | 0.218 | 0.217 | 22 | 9 |
| | C | 0.394 | 0.18 | 2.193 | 0.691 | 0.77 | 38 | 17 |
| | D | 0.258 | 0.135 | 1.91 | 0.654 | 0.66 | 77 | 40 |
| | E | -0.287 | 0.189 | -1.52 | 0.377 | 0 | 81 | 1 |
| | F | -0.432 | 0.189 | -2.291 | 0.297 | – | 96 | – |
| | rest | 0.097 | 0.185 | 0.527 | 0.656 | 0.535 | 27 | 23 |
| Chick fledging | W | -0.866 | 0.26 | -3.333 | 0.462 | – | 13 | – |
| | V | -0.489 | 0.221 | -2.214 | 0.611 | – | 18 | – |
| | U | -0.096 | 0.171 | -0.564 | 0.767 | – | 30 | – |
| | R | 0.391 | 0.187 | 2.088 | 0.909 | 1 | 11 | 14 |
| | P | 0.177 | 0.148 | 1.193 | 0.862 | 0.909 | 29 | 11 |
| | Q | 0.231 | 0.174 | 1.329 | 0.917 | 0.882 | 12 | 17 |
| | M | -0.478 | 0.26 | -1.84 | 0.615 | – | 13 | – |
| | L | 0.298 | 0.26 | 1.147 | 1 | 0.917 | 1 | 12 |
| | I | 0.207 | 0.119 | 1.742 | – | 0.887 | – | 62 |
| | J | 0.25 | 0.11 | 2.282 | – | 0.904 | – | 73 |
| | H | -0.949 | 0.354 | -2.681 | 0.4 | 0.5 | 5 | 2 |
| | C | -0.062 | 0.146 | -0.421 | 0.75 | 0.846 | 28 | 13 |
| | D | 0.328 | 0.107 | 3.075 | 0.941 | 0.923 | 51 | 26 |
| | E | -0.685 | 0.171 | -4.005 | 0.533 | – | 30 | – |
| | F | -0.769 | 0.177 | -4.344 | 0.5 | – | 28 | – |
| | rest | -0.117 | 0.174 | -0.671 | 0.882 | 0.583 | 17 | 11 |

**Table S2.** Predictors of edible-nest swiftlet clutch success for egg-hatching and chick-fledging. Analysis was run only for edible-nest swiftlets (clutch size=2 eggs) as black-nest swiftlets have single-egg clutches. Estimates are shown with their upper and lower 95% confidence intervals (calculated using 'glht' function from the R package 'multcomp v.1.4-18'). Abbreviations: SE – standard error, CIs – confidence intervals, var – variance.

| model | predictor | estimate | SE | lower CI | upper CI | Z-value | P |
|---|---|---|---|---|---|---|---|
| **Egg hatching** | intercept | 0.060 | 0.098 | -0.159 | 0.28 | 0.614 | |
| $N_{obs}$ = 313 clutches | distance from nearest entrance | -0.171 | 0.075 | -0.339 | -0.004 | -2.286 | 0.024 |
| | var (nest identity) | 0.101 | | | | | 0.012 |
| | var (wall identity) | 0.074 | | | | | 0.081 |
| | var (residual) | 0.814 | | | | | |
| **Chick fledging** | intercept | -0.023 | 0.125 | -0.303 | 0.256 | -0.186 | |
| $N_{obs}$= 131 clutches | distance from nearest entrance | -0.167 | 0.100 | -0.390 | 0.056 | -1.679 | 0.091 |
| | var (nest identity) | 0.010 | | | | | 1.000 |
| | var (wall identity) | 0.112 | | | | | 0.019 |
| | var (residual) | 0.859 | | | | | |

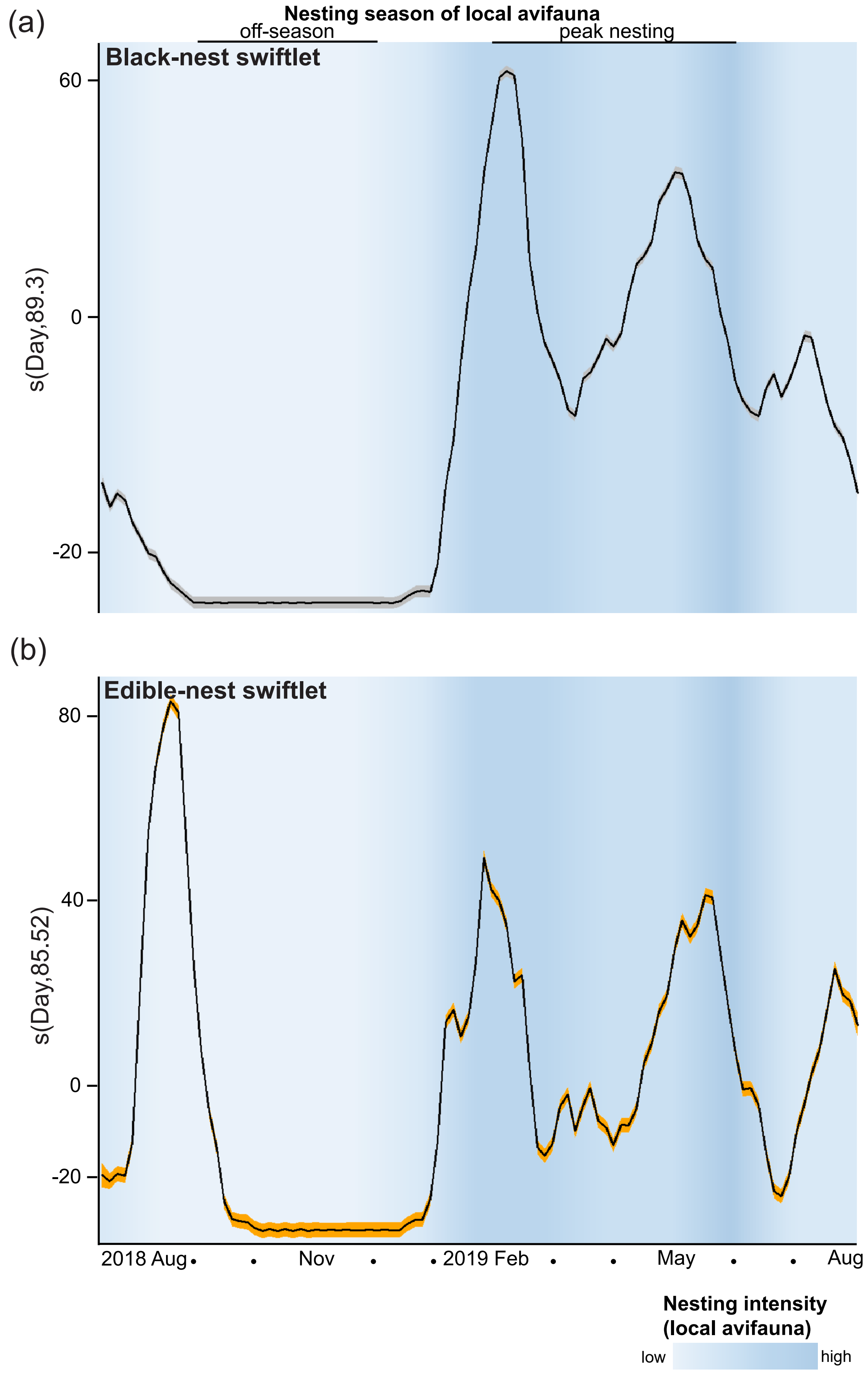


**FIGURE S1** Edible-nest swiftlets display an additional off-season breeding peak. Relationships between the number of eggs and time of year for (a) black-nest swiftlets and (b) edible-nest swiftlets are depicted (smoothers, black lines; 95% confidence bands, gray and orange shading). Y-axis labels provide estimated degrees of freedom of the smoothers. Nesting intensity of avifauna in Singapore is defined by Berman et al., 2022.
